# Prevalence of gastroduodenal ulcers/erosions in patients taking low-dose aspirin with either 15 mg/day of lansoprazole or 40 mg/day of famotidine: The OITA-GF study 2

**DOI:** 10.1186/1756-0500-6-116

**Published:** 2013-03-26

**Authors:** Akira Tamura, Kazunari Murakami, Junichi Kadota

**Affiliations:** 1 Internal Medicine 2, Oita University, Yufu, Japan; 2 General Medicine, Oita University, Yufu, Japan

**Keywords:** Aspirin, Gastroduodenal erosion, Gastroduodenal ulcer, Famotidine, Lansoprazole

## Abstract

**Background:**

The preventive effects of histamine 2 receptor antagonists vs. proton pump inhibitors on low-dose aspirin (LDA)-related gastroduodenal mucosal injury have not been fully investigated. We conducted a cross-sectional study to compare the prevalence of gastroduodenal ulcers or erosions in patients taking LDA with either 40 mg/day of famotidine or 15 mg/day of lansoprazole for at least three months.

**Methods:**

Of 84 eligible patients, two taking 40 mg/day of famotidine and four taking 15 mg/day of lansoprazole refused to undergo upper gastrointestinal endoscopy. Ultimately, we performed upper gastrointestinal endoscopy in 78 patients taking either 40 mg/day of famotidine (group F, n = 31) or 15 mg/day of lansoprazole (group L, n = 47). The prevalence of gastroduodenal ulcers or erosions and the magnitude of gastric mucosal injury evaluated using modified Lanza scores were compared between the two groups.

**Results:**

No patients in either group had gastroduodenal ulcers. Gastroduodenal erosions were more prevalent in group F than in group L (48.4% vs. 17.0%, p = 0.005). The modified Lanza scores (mean ± SD) were significantly higher in group F than in group L (0.9 ± 1.3 vs. 0.3 ± 0.7, p = 0.007). A multivariate logistic regression analysis showed that the use of lansoprazole was negatively associated with gastroduodenal erosions.

**Conclusions:**

This study suggests that 15 mg/day of lansoprazole may be more effective in preventing the development of LDA-related gastroduodenal erosions than 40 mg/day of famotidine. The preventive effects of these two regimens on the development of LDA-related gastroduodenal ulcers require further investigation.

## Background

Low-dose aspirin (LDA) (75 – 325 mg/day), which is widely used for the primary and secondary prevention of cardiovascular events and the prevention of coronary stent thrombosis [1,2], is associated with a 2- to 4-fold increased risk of upper gastrointestinal complications, such as gastroduodenal ulcers and bleeding [3,4]. Therefore, it is clinically important to prevent gastroduodenal mucosal injury in patients talking LDA. Proton pump inhibitors (PPIs) are considered to be the preferred agents for prophylaxis of LDA-related gastroduodenal mucosal injury [5]. Famotidine, a histamine 2 receptor antagonist (H2RA), has also been reported to suppress the development of LDA-related gastroduodenal ulcers [6]. One prospective, randomized, comparative study [7] examined the effects of H2RAs vs. PPIs on the primary prevention of LDA-related gastric mucosal injury and showed that 15 mg/day of lansoprazole is more effective in preventing LDA-related gastric mucosal injury than 40 mg/day of famotidine. However, in that study, the duration of treatment was only seven days. Another prospective, randomized, comparative study [8] examined the effects of H2RAs vs. PPIs on the secondary prevention of LDA-related dyspeptic or bleeding gastroduodenal ulcers and erosions and showed that 80 mg/day of famotidine is inferior to 20 mg/day of pantoprazole in the secondary prevention of recurrent dyspeptic or bleeding gastroduodenal ulcers and erosions. However, in that study, follow-up endoscopic examinations were performed only in patients with dyspepsia or bleeding (melena or hematemesis). It is well known that LDA-related gastroduodenal mucosal injury is often asymptomatic [9,10]. Therefore, whether there are distinct differences in the effects of H2RAs vs. PPIs on the primary and secondary prevention of LDA-related gastroduodenal mucosal injury has not been fully clarified. We previously demonstrated that PPIs, but not H2RAs, are negatively and independently associated with gastroduodenal ulcers or erosions in asymptomatic patients taking LDA [11]. In the present study, we compared the prevalence of gastroduodenal ulcers or erosions in patients taking LDA with either 40 mg/day of famotidine or 15 mg/day of lansoprazole for at least three months.

## Methods

This cross-sectional study was conducted between April 2011 and June 2012 at Oita University Hospital in accordance with the Declaration of Helsinki and its amendments. The study protocol was approved by the ethics committee of Oita University Hospital, and written informed consent was obtained from all patients before enrollment.

### Study population

Eligible inpatients or outpatients of the Cardiology Division of Oita University Hospital who were taking LDA with either 40 mg/day of famotidine or 15 mg/day of lansoprazole for at least three months were prospectively enrolled in this study, unrelated to the presence or absence of gastrointestinal symptoms. The inclusion criteria were as follows: age > 20 years; no history of surgery for esophageal, gastric or duodenal diseases; neither known gastrointestinal bleeding nor gastroduodenal ulcers within the previous 12 months; neither acute coronary syndrome nor stroke within the previous three months; an absence of severe chronic heart failure (New York Heart Association functional class IV); a lack of use of steroids or nonsteroidal anti-inflammatory drugs; and an absence of malignant diseases.

### Data collection

The following demographic data were collected: age, gender, body mass index, the presence or absence of current smoking, daily alcohol drinking (unrelated to the amount of alcohol), hypertension, dyslipidemia and diabetes mellitus, a history of cardiovascular disease, gastroduodenal ulcers or eradication of *Helicobacter pylori* (*H. pylori*), gastrointestinal symptoms, the *H. pylori* infection status and current medications. Current smoking was defined as daily or non-daily smoking at the time of study entry. Hypertension was defined as a systolic blood pressure ≥140 mmHg, a diastolic blood pressure ≥90 mmHg or treatment with antihypertensive medications. Dyslipidemia was defined as a serum low-density lipoprotein cholesterol level ≥140 mg/dl, a high-density lipoprotein cholesterol level <40 mg/dl, a triglyceride level ≥150 mg/dl or treatment with lipid-lowering drugs. Diabetes mellitus was defined as a fasting plasma glucose level ≥126 mg/dl, a plasma glucose level ≥200 mg/dl at two hours after a 75-g glucose load or treatment with hypoglycemic agents. Gastrointestinal symptoms (reflux, abdominal pain and indigestion) were evaluated using the gastrointestinal symptom rating scale [12] immediately before esophagogastroduodenal endoscopy. The presence or absence of *H. pylori* infection was examined using a urine-based enzyme-linked immunosorbent assay (Otsuka Pharmaceutical, Tokyo, Japan) [13,14].

### Endoscopic examinations

Esophagogastroduodenal endoscopy was performed without cessation of LDA. An ulcer was defined as a mucosal defect having a significant depth, measuring at least 3 mm over its longest diameter. An erosion was defined as a mucosal defect measuring less than 3 mm. The magnitude of gastric mucosal injury was assessed using the modified Lanza score [15] (Table 1). The evaluations were conducted by experienced endoscopists who were blinded to all clinical data.

**Table 1:** Modified Lanza scores

**Score**	**Findings**
0	No hemorrhage or erosions observed
1	One or two hemorrhages or erosions observed in one gastric area
2	Three to five hemorrhages or erosions observed in one gastric area
3	Hemorrhages or erosions observed in two gastric areas or six or more hemorrhages or erosions observed in one gastric area, with the total number not exceeding 10 in the entire stomach
4	Hemorrhages or erosions observed in three or more gastric areas or 11 or more hemorrhages or erosions observed in the entire stomach
5	Gastric ulcers

### Statistical analysis

Continuous data are expressed as the mean ± SD. Comparisons of continuous variables between two groups were made using the unpaired *t*-test or the Mann–Whitney *U*-test. Comparisons of categorical variables between two groups were made using the Fisher’s exact test or the chi-square test. A multivariate logistic regression analysis was performed to determine whether the use of 15 mg/day of lansoprazole was independently associated with gastroduodenal erosions. A p value of <0.05 was considered to be statistically significant. All analyses were performed using the IBM SPSS Statistics 20 software package (International Business Machines Corp., New York, USA).

## Results

**Figure 1:**
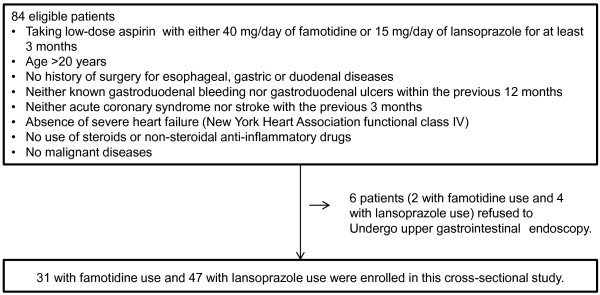
Flow chart of the study cohort.

Between April 2011 and June 2012, there were 84 eligible patients taking LDA with either 40 mg/day of famotidine or 15 mg/day of lansoprazole for at least three months. Of the 84 patients, two taking 40 mg/day of famotidine and four taking 15 mg/day of lansoprazole refused to undergo upper gastrointestinal endoscopy. Ultimately, 78 patients taking LDA with either 40 mg/day of famotidine (group F, n = 31) or 15 mg/day of lansoprazole (group L, n = 47) for at least three months were enrolled in the present study (Figure 1).

The patient characteristics are shown in Table 2. There were no significant differences in patient characteristics between the two groups.

**Table 2:** Patient characteristics

	**Group F (n = 31)**	**Group L (n = 47)**	**p value**
Age (years)	72.0 ± 9.3	74.6 ± 8.8	0.22
Male	26 (83.9%)	32 (68.1%)	0.19
Body mass index (kg/m^2^)	23.8 ± 3.4	24.3 ± 3.6	0.50
Hypertension	25 (80.6%)	44 (93.6%)	0.14
Diabetes mellitus	9 (29.0%)	18 (38.3%)	0.47
Current smoking	0 (0%)	2 (4.3%)	0.52
Daily alcohol drinking	12 (38.7%)	13 (27.7%)	0.33
Coronary artery disease	26 (83.9%)	43 (91.5%)	0.47
Prior percutaneous coronary intervention	25 (80.6%)	39 (83.0%)	1.0
Prior coronary bypass surgery	1 (3.2%)	2 (4.3%)	1.0
Prior stroke	4 (12.9%)	4 (8.5%)	0.71
Atrial fibrillation	6 (19.4%)	7 (14.9%)	0.76
History of gastroduodenal ulcer	5 (16.1%)	9 (19.1%)	0.77
History of eradication of *H*. *pylori*	3 (9.7%)	5 (10.6%)	1.0
Gastrointestinal symptom rating scale			
Reflux symptom score	1.4 ± 0.6	1.3 ± 0.6	0.19
Abdominal pain score	1.4 ± 0.7	1.2 ± 0.5	0.32
Indigestion score	1.4 ± 0.6	1.6 ± 0.6	0.24
Positive urinary *H*. *pylori* antibodies	15 (48.4%)	22 (46.8%)	1.0
Duration of lansoprazole or famotidine treatment			0.76
<1 year	7 (14.9%)	6 (19.4%)	
≥1 year(s)	40 (85.1%)	25 (80.6%)	
Dose of aspirin			1.0
81 mg/day	1 (3.2%)	2 (4.3%)	
100 mg/day	30 (96.8%)	45 (95.7%)	
Type of aspirin			1.0
Enteric-coated formulation	30 (96.8%)	45 (95.7%)	
Buffered formulation	1 (3.2%)	2 (4.3%)	
Other antiplatelet agents	12 (38.7%)	17 (36.2%)	1.0
Warfarin	8 (25.8%)	8 (17.0%)	0.40
Dabigatran	2 (7.4%)	2 (4.3%)	0.62
Nitrates	10 (32.3%)	16 (34.0%)	1.0
Calcium antagonists	14 (45.2%)	26 (55.3%)	0.49
Angiotensin-converting enzyme inhibitors	3 (9.7%)	4 (8.5%)	1.0
Angiotensin receptor blockers	14 (45.2%)	30 (63.8%)	0.16
Statins	27 (87.1%)	42 (89.4%)	1.0

The data are presented as the mean ± SD or number (%).

**Figure 2:**
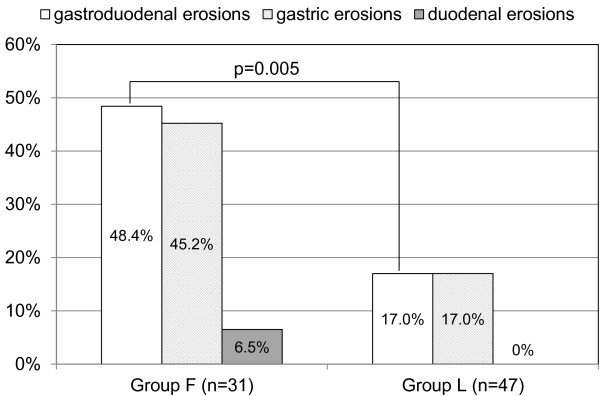
Comparison of the prevalence of gastroduodenal erosions between patients taking 40 mg/day of famotidine (group F) and those taking 15 mg/day of lansoprazole (group L).

No patients had gastroduodenal ulcers. Gastroduodenal erosions were more prevalent in group F than in group L (48.4% vs. 17.0%, p = 0.005) (Figure 2).

The modified Lanza scores (mean ± SD) were significantly higher in group F than in group L (0.9 ± 1.3 vs. 0.3 ± 0.7, p = 0.007). After adjusting for confounding factors, including age, male gender, body mass index, current smoking, daily alcohol drinking, a history of gastroduodenal ulcers and positive urinary *H. pylori* antibodies, the use of lansoprazole was found to be negatively associated with gastroduodenal erosions (odds ratio 0.18, 95% confidence interval 0.06-0.59, p = 0.005).

## Discussion

### Primary findings

The primary findings of the present study are as follows: (1) gastroduodenal erosions were less prevalent in patients taking LDA with 15 mg/day of lansoprazole than in those taking LDA with 40 mg/day of famotidine; (2) no patients in either group had gastroduodenal ulcers; and (3) the multivariate logistic regression analysis showed that the use of 15 mg/day of lansoprazole was negatively associated with gastroduodenal erosions.

### Previous studies

Although PPIs are considered to be the preferred agents for prophylaxis of LDA-related gastroduodenal mucosal injury [5], there are only two prospective, randomized, comparative studies examining the effects of H2RAs vs. PPIs on the prevention of LDA-related gastric mucosal injury [7,8]. Nishio et al. [7] randomly administered 100 mg/day of aspirin, 100 mg/day of aspirin plus 40 mg/day of famotidine or 100 mg/day of aspirin plus 15 mg/day of lansoprazole for seven days in a crossover fashion in 15 *H. pylori*-negative healthy volunteers and compared the magnitude of gastric mucosal injury among the three regimens using modified Lanza scores. The results showed that the modified Lanza scores were significantly lower in the 100 mg/day of aspirin plus 15 mg/day of lansoprazole group than in the other two groups, suggesting that 15 mg/day of lansoprazole may be more effective in preventing LDA-related gastric mucosal injury than 40 mg/day of famotidine. However, in that study, the subjects were *H. pylori*-negative healthy young volunteers, and the duration of treatment was only seven days. Ng et al. [8] compared the recurrence rate of LDA-related dyspeptic or bleeding gastroduodenal ulcers and erosions occurring within 48 weeks between 65 patients receiving famotidine (80 mg/day) treatment and 65 patients receiving pantoprazole (20 mg/day) treatment and showed that the recurrence rate of dyspeptic or bleeding gastroduodenal ulcers and erosions was lower in the latter group than in the former group (0% vs. 9.2%, p = 0.03). However, in that study, follow-up endoscopic examinations were performed only in patients with dyspepsia or bleeding (melena or hematemesis). Therefore, the exact effects of H2RAs vs. PPIs on the primary and secondary prevention of LDA-related gastroduodenal mucosal injury have not been fully clarified.

### The present study

In the present study, we compared the prevalence of gastroduodenal ulcers or erosions in patients taking LDA with either 40 mg/day of famotidine or 15 mg/day of lansoprazole for at least three months and found that gastroduodenal erosions were less prevalent in patients taking LDA with 15 mg/day of lansoprazole than in those taking LDA with 40 mg/day of famotidine. This result suggests that 15 mg/day of lansoprazole may be more effective in preventing the development of LDA-related gastroduodenal erosions than 40 mg/day of famotidine. Gastroduodenal erosions are the most readily observable finding of initial gastroduodenal mucosal injury following aspirin administration [16]. Gastric acid plays an important role in the development of LDA-related gastroduodenal mucosal injury, and the severity of LDA-related gastric mucosal injury is associated with gastric acidity [7,17]. Because PPIs are more effective in suppressing the secretion of gastric acid than H2RAs [7,17,18], the findings of the present study are reasonable. However, it should be noted that, in the present study, the mean modified Lanza score among the patients taking 40 mg/day of famotidine was low and that the difference in the scores between the two groups was small. In the present study, none of the patients taking either 40 mg/day of famotidine or 15 mg/day of lansoprazole had gastroduodenal ulcers. Yeomans et al. [10] reported that gastroduodenal ulcers were observed in 10.7% of 187 patients taking LDA without gastroprotective medications. In the OBERON trial, which compared the occurrence of peptic ulcers over 26 weeks among patients taking LDA with those taking a placebo, 20 mg/day of esomeprazole or 40 mg/day of esomeprazole, peptic ulcers developed in 7.4% of 805 placebo recipients (an intention-to-treat analysis) [19]. Given that, in the present study, 82.1% of the patients had no history of gastroduodenal ulcers and that none of the patients in the famotidine group had gastroduodenal ulcers, 40 mg/day of famotidine may be as effective as 15 mg/day of lansoprazole in the primary prevention of LDA-related gastroduodenal ulcers. The preventive effects of 40 mg/day of famotidine vs. 15 mg/day of lansoprazole on the development of LDA-related gastroduodenal ulcers require further investigation.

### The clinical significance of gastroduodenal erosions in patients taking LDA

The prevention of gastroduodenal erosions is considered to be clinically important in patients taking LDA for the following reasons. First, in patients taking LDA, some gastroduodenal erosions may bleed. It has been shown that in 16% to 23% of patients with upper gastrointestinal bleeding, gastroduodenal erosions are the cause of the bleeding [20,21]. Furthermore, gastric erosions have been reported to be the second most common source of gastrointestinal bleeding after acute myocardial infarction [22] in which the use of LDA is essential for secondary prevention. Second, some gastroduodenal erosions may develop into gastroduodenal ulcers in patients taking LDA. It has been reported that one-sixth of gastroduodenal erosions become gastroduodenal ulcers after 17 years [23]. Furthermore, the presence of gastroduodenal erosions has been reported to increase the risk of developing gastroduodenal ulcers in nonsteroidal anti-inflammatory drug users [24].

### The strength and limitations of the present study

The strength of the present study is the sufficient sample size to detect significant differences in the prevalence of gastroduodenal erosions between the two groups. A post hoc power determinant analysis showed that the present study has an estimated power of 85% with a two-sided alpha risk of 0.05. On the other hand, the present study has a few limitations. First, this study had a cross-sectional design, which could potentially carry a risk of reverse causation. The duration of lansoprazole and famotidine treatment was not identical between the two groups. In addition, we did not investigate why either 40 mg/day of famotidine or 15 mg/day of lansoprazole was selected for each patient. Therefore, prospective, randomized, longitudinal studies are needed to clarify whether 15 mg/day of lansoprazole is indeed more effective in preventing LDA-related gastroduodenal erosions than 40 mg/day of famotidine. Second, we did not investigate the mechanisms of the lower prevalence of gastroduodenal erosions observed in the lansoprazole group. Third, larger doses of famotidine and lansoprazole may be more effective in preventing LDA-related gastroduodenal erosions. Further investigations are required to clarify the associations between the doses of famotidine and lansoprazole and their preventive effects on the development of LDA-related gastroduodenal erosions.

## Conclusion

The results of the present study suggest that 15 mg/day of lansoprazole may be more effective in preventing LDA-related gastroduodenal erosions than 40 mg/day of famotidine. Elucidating the preventive effects of these two regimens on the development of LDA-related gastroduodenal ulcers, however, still requires further investigation.

## Appendix

OITA-GF Study 2 investigators: Y. Goto, K. Torigoe, Y. Kawano, K. Shinozaki, M. Kotoku, Internal Medicine 2, Oita University; Y. Hirashita, R. Ogawa, O. Matsunari, T. Abe, S. Shiota, K. Mizukami, Y. Nakagawa, M. Uchida, T. Okimoto, M. Kodama, General Medicine, Oita University.

## Abbreviations

H. pylori: Helicobacter pylori; H2RA: Histamine 2 receptor antagonist; LDA: Low-dose aspirin; PPI: Proton pump inhibitor

## Competing interests

The authors declare that they have no competing interests.

## Authors’ contributions

AT, KM and JK were involved in the design of the study, the review of the literature and the writing of the manuscript. The OITA-GF2 investigators were involved in the data collection. All authors read and approved the manuscript.

## Authors’ information

The investigators of the OITA-GF study 2 are listed in Appendix.
